# Genomic characterization of *Kerstersia gyiorum* SWMUKG01, an isolate from a patient with respiratory infection in China

**DOI:** 10.1371/journal.pone.0214686

**Published:** 2019-04-12

**Authors:** Ying Li, Min Tang, Guangxi Wang, Chengwen Li, Wenbi Chen, Yonghong Luo, Jing Zeng, Xiaoyan Hu, Yungang Zhou, Yan Gao, Luhua Zhang

**Affiliations:** 1 Department of Immunology, School of Basic Medical Sciences, Southwest Medical University, Luzhou, Sichuan, China; 2 Department of Pathogenic Biology, School of Basic Medical Sciences, Southwest Medical University, Luzhou, Sichuan, China; 3 Department of Physiology, School of Basic Medical Sciences, Southwest Medical University, Luzhou, Sichuan, China; Cornell University, UNITED STATES

## Abstract

**Background:**

The Gram-negative bacterium *Kerstersia gyiorum*, a potential etiological agent of clinical infections, was isolated from several human patients presenting clinical symptoms. Its significance as a possible pathogen has been previously overlooked as no disease has thus far been definitively associated with this bacterium. To better understand how the organism contributes to the infectious disease, we determined the complete genomic sequence of *K*. *gyiorum* SWMUKG01, the first clinical isolate from southwest China.

**Results:**

The genomic data obtained displayed a single circular chromosome of 3, 945, 801 base pairs in length, which contains 3, 441 protein-coding genes, 55 tRNA genes and 9 rRNA genes. Analysis on the full spectrum of protein coding genes for cellular structures, two-component regulatory systems and iron uptake pathways that may be important for the success of the bacterial survival, colonization and establishment in the host conferred new insights into the virulence characteristics of *K*. *gyiorum*. Phylogenomic comparisons with *Alcaligenaceae* species indicated that *K*. *gyiorum* SWMUKG01 had a close evolutionary relationships with *Alcaligenes aquatilis* and *Alcaligenes faecalis*.

**Conclusions:**

The comprehensive analysis presented in this work determinates for the first time a complete genome sequence of *K*. *gyiorum*, which is expected to provide useful information for subsequent studies on pathogenesis of this species.

## Introduction

*Kerstersia gyiorum* is a Gram-negative coccobacillus that is occasionally isolated from clinical samples of human infections. On nutrient agar, colonies of *K*. *gyiorum* are usually characterized by spreading edge morphology, exhibiting flat or slightly convex with smooth margins [1–3]. The word ‘gyiorum’, meaning ‘from the limbs’, was given as a species name by Coenye *et al*., since the organism was primarily isolated from lower-extremity wounds. The novel genus *Kerstersia*, initially described by Coenye *et al*., is grouped in the family *Alcaligenaceae* along with *Alcaligenes*, *Achromobacter*, *Bordetella*, and *Pigmentiphaga* spp. [1]. *Kerstersia* members resemble *Alcaligenes faecalis* phenotypically, except that isolates of *Kerstersia* are oxidase-negative and do not produce a fruity odor.

The initial publication describing *K*. *gyiorum* by Coenye *et al*. in 2003 reported six isolates recovered from leg wounds, sputum, and feces. After 2012, case reports documenting *Kerstersia* spp. infection began to emerge again. Since then, there have been one case of infection with a second species of *Kerstersia*, *Kerstersia similis* [4] and 12 publications describing 14 cases of patients with various diseases infected with *K*. *gyiorum* [2, 3, 5–13]. Among them, seven cases were associated with chronic otitis media [2, 5, 6, 8, 11, 13], three with chronic leg wound [2, 7, 10]. Additionally, *K*. *gyiorum* was also reported to be isolated from patients with chronic tracheostomy [3], chronic osteomyelitis [12], or urinary tract infection [9]. In fact, it is difficult to distinguish *K*. *gyiorum* from other microorganisms using conventional methods, such as traditional biochemical tests and automated identification systems, which may lead to *K*. *gyiorum* being identified incorrectly or unsuccessfully in the past in most clinical laboratories [12]. The potentially clinical importance of *K*. *gyiorum* may therefore be overlooked. For the current, with the development of (matrix-assisted laser desorption/ionization time-of-flight mass spectrometry) MALDI–TOF MS and 16S rRNA gene sequencing, *K*. *gyiorum* is expected to be identified in laboratories more accurately.

As previous reports indicated, other bacterial species were co-isolated with *K*. *gyiorum* from specimens in most cases [2, 3, 6, 9]. It was suggested that *K*. *gyiorum* has an affinity towards causing chronic mixed infections in patients [6]. However, little is known regarding how much of the disease process can specifically be attributed to *K*. *gyiorum* because of a lack of pathogenesis-related information on this organism in the literature. Reported cases of human *Kerstersia* infections showed that the patients gained remission when treated with an antimicrobial agent to which this organism was susceptible [2, 8, 9]. These observations indicate that *K*. *gyiorum* could contribute significantly as a possible aetiology. To better understand how the organism contributes to infections, virulence factors and pathogenic mechanisms of *K*. *gyiorum* need to be investigated. Whole genome sequencing represents a valuable approach in an in-depth exploration of potential virulence factors and may answer important questions concerning the evolution of this bacterium [14]. Though a draft genome of *K*. *gyiorum* was announced by Greninger *et al* [15], a comprehensive genome analysis of this organism is still not available to date. For this study, we presented a complete genome sequence of recently identified isolate *K*. *gyiorum* SWMUKG01, which was recovered from the sputum of a patient with respiratory infection. We reported and analyzed the complete genome sequence of *K*. *gyiorum* SWMUKG01, accompanied by a detailed annotation of its genome organization and expression strategy, with an emphasis on the investigation of genes and operons related to potential virulence factors.

## Material and methods

### Bacterial strain and growth conditions

The *K*. *gyiorum* strain SWMUKG01 was recovered from the sputum of a 70-year-old female patient of respiratory infection with a history of tracheotomy and epilepsy in May, 2018 from the affiliated hospital of Southwest Medical University. The isolate SWMUKG01 was identified as *K*. *gyiorum* by MALDI-TOF MS and 16S rRNA gene sequencing. For genomic DNA extraction, one colony of *K*. *gyiorum* SWMUKG01 was transferred into 5 ml Tryptic Soy Broth (TSB, Difco Laboratories) and cultured at 37°C. After an overnight incubation, the culture was diluted 1:100 into fresh TSB for sub-cultivation until mid-exponential growth phase was reached. The bacterial cells were collected by centrifugation at 5000 g for 10 min.

### DNA extraction and genome sequencing

Total genomic DNA of *K*. *gyiorum* SWMUKG01 was extracted using Rapid Bacterial Genomic DNA Isolation Kit (Sangon Biotech, Shanghai, China) according to the manufacturer’s protocol. The genomic DNA was sent to Sangon Biotech (Shanghai, China) for *de novo* whole genome sequencing. A combination of HiSeq 2500 Sequencer (Illumina, San Diego, CA, USA) and the PacBio RSII platforms (Pacific Biosciences, Menlo Park, CA, USA) was employed for whole genome sequencing. *De novo* genome assembly of filtered reads was conducted using the Hierarchical Genome Assembly Process workflow (HGAP, v3; Pacific Biosciences) [16].

### Sequence analysis

Protein coding sequences (CDSs), tRNAs and rRNAs were predicted in *K*. *gyiorum* SWMUKG01 complete genome with Prokka [17]. BLAST [18] was employed for annotation based on the sequence similarity of CDS against the Cluster of Orthologous Groups of proteins (COG) [19, 20], Kyoto Encyclopedia of Genes and Genomes (KEGG) [21], SwissProt [22], NR, and Gene Ontology (GO) databases [23]. The genome circular map was performed with the program CGview [24]. BLAST was employed to align the gene sequences against the Comprehensive Antibiotic Resistance Database (CARD) [25], and the description of the best hit (with the highest alignment length percentage and match identity) was assigned as the annotation of predicted gene. Virulence factors were investigated by BLAST against the virulence factor database (VFDB) (*E* < 1e^-5^) [26]. Genomic islands were annotated using IslandViewer 4 (http://www.pathogenomics.sfu.ca/islandviewer/) [27].

### Phylogenetic analysis

For phylogenetic and comparative analysis, the genome sequences of the closest genetic relatives of *K*. *gyiorum* SWMUKG01 were obtained from the NCBI including *K*. *gyiorum* CG1 (NZ_LBNE00000000.1), *A*. *faecalis* ZD02 (NZ_CP013119.1), *Alcaligenes aquatilis* BU33N (NZ_CP022390.1), *Achromobacter xylosoxidans* NCTC10807 (NZ_LN831029.1), *Achromobacter denitrificans* USDA-ARS-USMARC-56712 (NZ_CP013923.1), *Achromobacter insolitus* FDAARGOS_88 (NZ_CP026973.1), *Bordetella bronchiseptica* 253 (NC_019382.1), *Bordetella pertussis* Tohama I (NC_002929.2), *Bordetella holmesii* ATCC 51541 (NZ_CP007494.1), *Bordetella hinzii* ASM107827v1 (NZ_CP012076.1), *Bordetella petrii* ASM6720v1 (NC_010170.1), *Bordetella parapertussis* Bpp5 (NC_018828.1), *Bordetella avium* 197N (NC_010645.1), *Bordetella trematum* H044680328 (NZ_LT546645.1), and *Bordetella pseudohinzii* HI4681 (NZ_CP016440.1). A phylogenetic tree based on the 16S rRNA gene sequences was generated using the tool FastTree [28]. To identify unique and conserved genes, the genome sequences of *K*. *gyiorum* SWMUKG01 and selected strains were compared using Pan-Genomes Analysis Pipeline (PGAP) [29], with which the genes shared by all genomes were collected, concatenated and aligned. The alignment of the conserved genes was used for the construction of neighbor joining tree using PGAP [29]. The species identification was also performed by average nucleotide identity (ANI) analysis between the isolate and strains shown in Table 1 using JSpeciesWS (http://jspecies.ribohost.com/jspeciesws/#analyse).

**Table 1:** General features of whole genomes of *K*. *gyiorum* (SWMUKG01), *K*. *gyiorum* (CG1), *A*. *faecalis* (ZD02), *A*. *aquatilis* (BU33N), *A*. *xylosoxidans* (NCTC10807), *A*. *insolitus* (FDAARGOS_88), *B*. *bronchiseptica* (253), *B*. *pertussis* (Tohama I) and *B*. *parapertussis* (Bpp5).

GenBank accession No.	CP033936	LBNE00000000	NZ_CP013119	NZ_CP022390	NZ_LN831029	NZ_CP026973	NC_019382	NC_002929	NC_018828
Strain	SWMUKG01	CG1	ZD02	BU33N	NCTC10807	FDAARGOS_88	253	Tohama I	Bpp5
Total length (bp)	3, 945, 801	3, 942, 939	4, 233, 756	3, 838, 399	6, 813, 182	6, 523, 893	5, 264, 383	4, 086, 189	4, 887, 379
GC content	62.0%	62.4%	56.8%	56.1%	67.4%	64.9%	68.1%	67.7%	67.8%
Number of CDSs	3521	3511	3785	3407	6087	6297	4781	3425	4184
Ribosome RNA									
16S rRNA	3	1	3	1	3	4	3	3	3
23S rRNA	3	1	3	1	3	4	3	3	3
5S rRNA	3	1	3	1	4	5	3	3	3
Number of tRNA	55	50	57	54	57	58	54	51	54

### Data availability

All relevant data are within the paper and its Supporting Information files. The genome sequence data of *K*. *gyiorum* SWMUKG01 were deposited in GenBank with the accession number CP033936. Raw sequencing data were deposited under a BioProject with accession number PRJNA497911.

### Ethics statement

The current study was approved by the Ethics Committee of Southwest Medical University (Sichuan, China). Written informed consent was exempted, since this retrospective study mainly focused on bacteria and patient intervention was not required.

## Results and discussions

### General features of the genome

**Fig 1:**
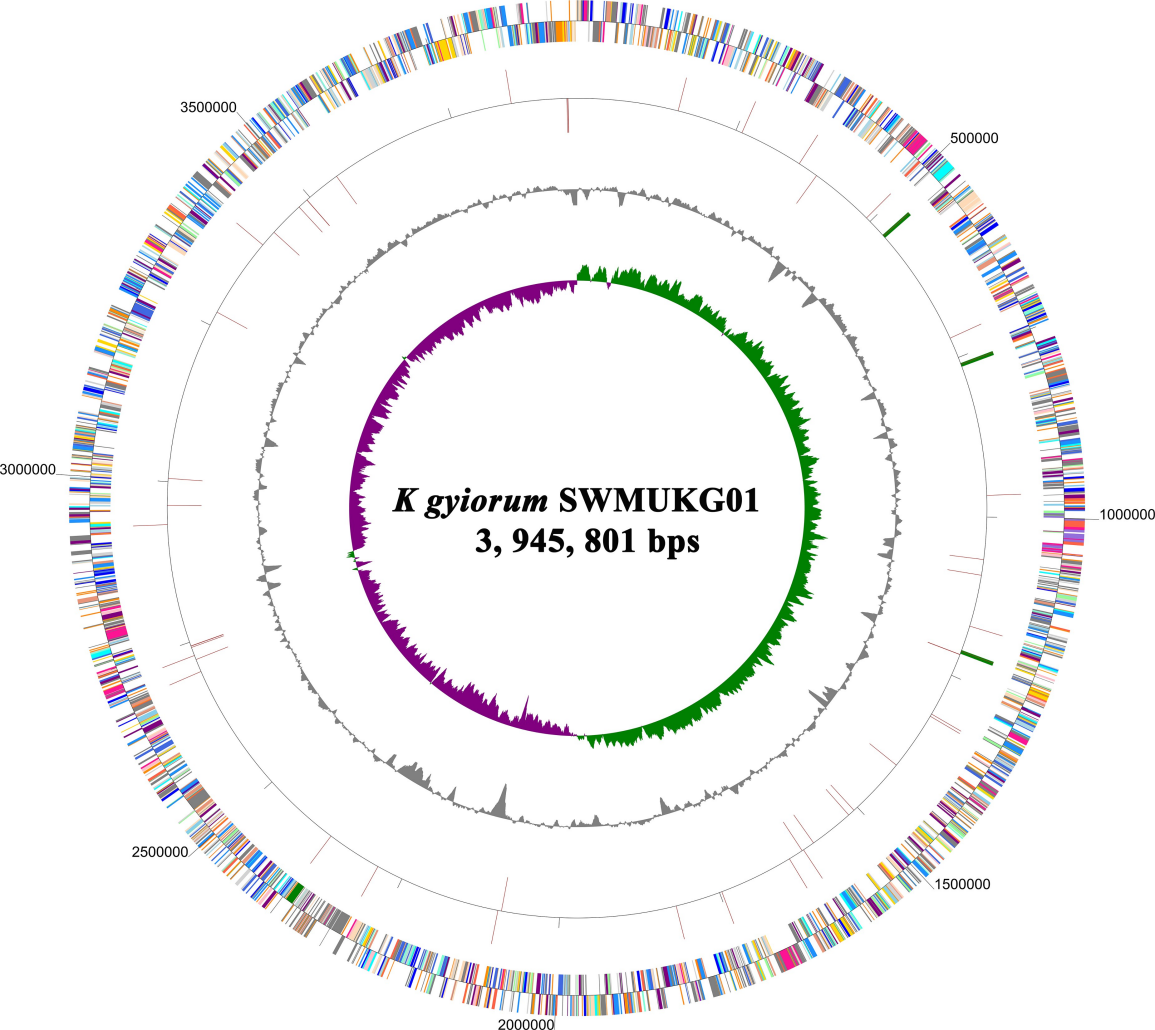
Circular map of the complete genome of *K*. *gyiorum* SWMUKG01. Circles from outside to inside: CDS on the forward strand (1) and CDS on the reverse strand (2), belonging to different COG categories were indicated by different colors; rRNA and tRNA genes (3); GC content (4); GC skew with + value (green) and–value (purple) (5).

The genome of *Kerstersia gyiorum* SWMUKG01 is composed of 3, 945, 801 base pairs (bps) with a single circular chromosome (Fig 1), which showed 95% coverage and 99% identity with *K*. *gyiorum* CG1. However, in CG1, the genome is comprised of one plasmid in addition to a circular chromosome. The putative replication origin (*oriC*) of SWMUKG01 chromosome was identified to be located from 3, 914, 889 to 3, 915, 821 bp, with the web-based system Ori-Finder [30]. The chromosome encodes 3520 predicted genes with an average length of 994 bps, which account for 88.72% of the whole chromosome in sum. The entire SWMUKG01 chromosome contains 55 tRNA genes and 9 rRNA genes. Global characterizations of the SWMUKG01 genome are compared to those of strains CG1 (*K*. *gyiorum*), ZD02 (*Alcaligenes faecalis*), BU33N (*A*. *aquatilis*), NCTC10807 (*A*. *xylosoxidans*), FDAARGOS_88 (*A*. *insolitus*), 253 (*B*. *bronchiseptica*), Tohama I (*B*. *pertussis*), and Bpp5 (*B*. *parapertussis*) (Table 1).

### Functional classification of *K*. *gyiorum* SWMUKG01

Orthologs are thought to retain the same function during evolution. Therefore, the identification of orthologs contributes to the prediction of gene functions in a newly identified species. In this study, NCBI COG database was employed for genome-scale analysis of protein function prediction in *K*. *gyiorum* SWMUKG01. Of the 3441 protein-coding genes in SWMUKG01, 2801 were categorized into 23 COG functional codes (S1 Fig, S1 Table), but 640 were not assigned. The majority of protein-coding genes were involved in basic cellular functions, such as metabolism, transcription and translation, 34.4% of genes have unknown function, including “general function prediction only,” “Function unknown,” and the genes not assigned (S2 Table).

### Phylogenetic analysis

**Fig 2:**
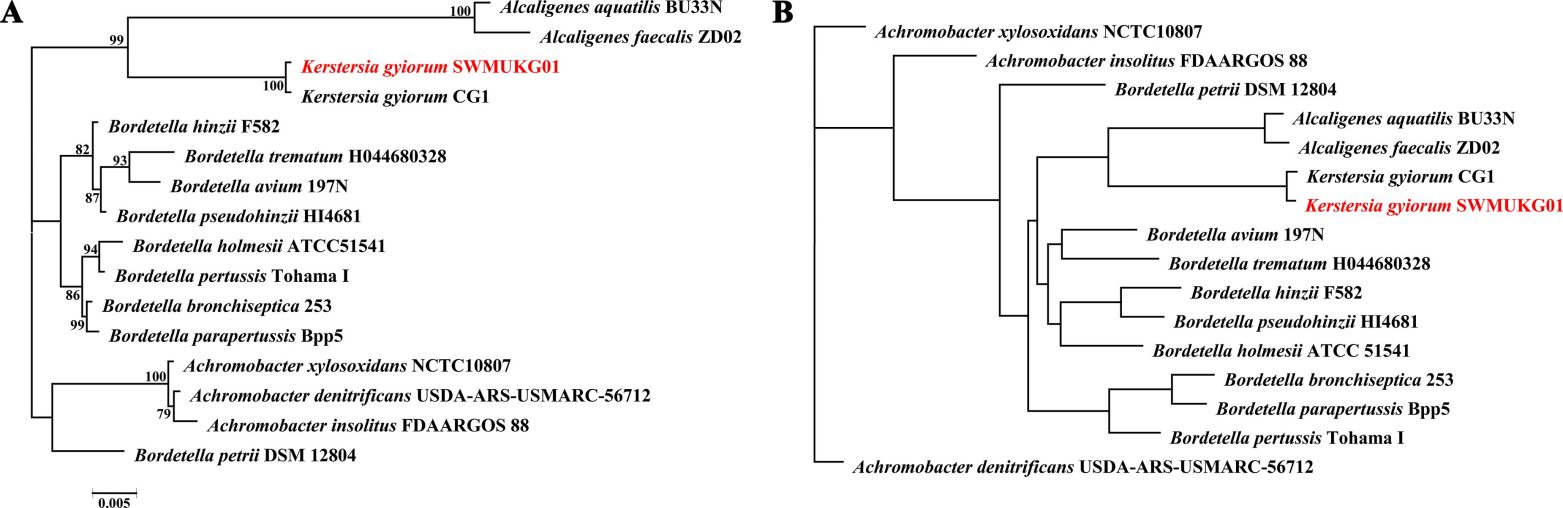
Phylogenetic trees of *K*. *gyiorum* SWMUKG01 and other closely related bacteria. **(A)** The phylogenetic tree was constructed using FastTree based on the 16S rRNA gene sequences. **(B)** The phylogenetic tree was constructed using PGAP based on 880 randomly concatenated, core genes in the genomes of *K*. *gyiorum* SWMUKG01 and 15 other closely related bacteria.

**Fig 3:**
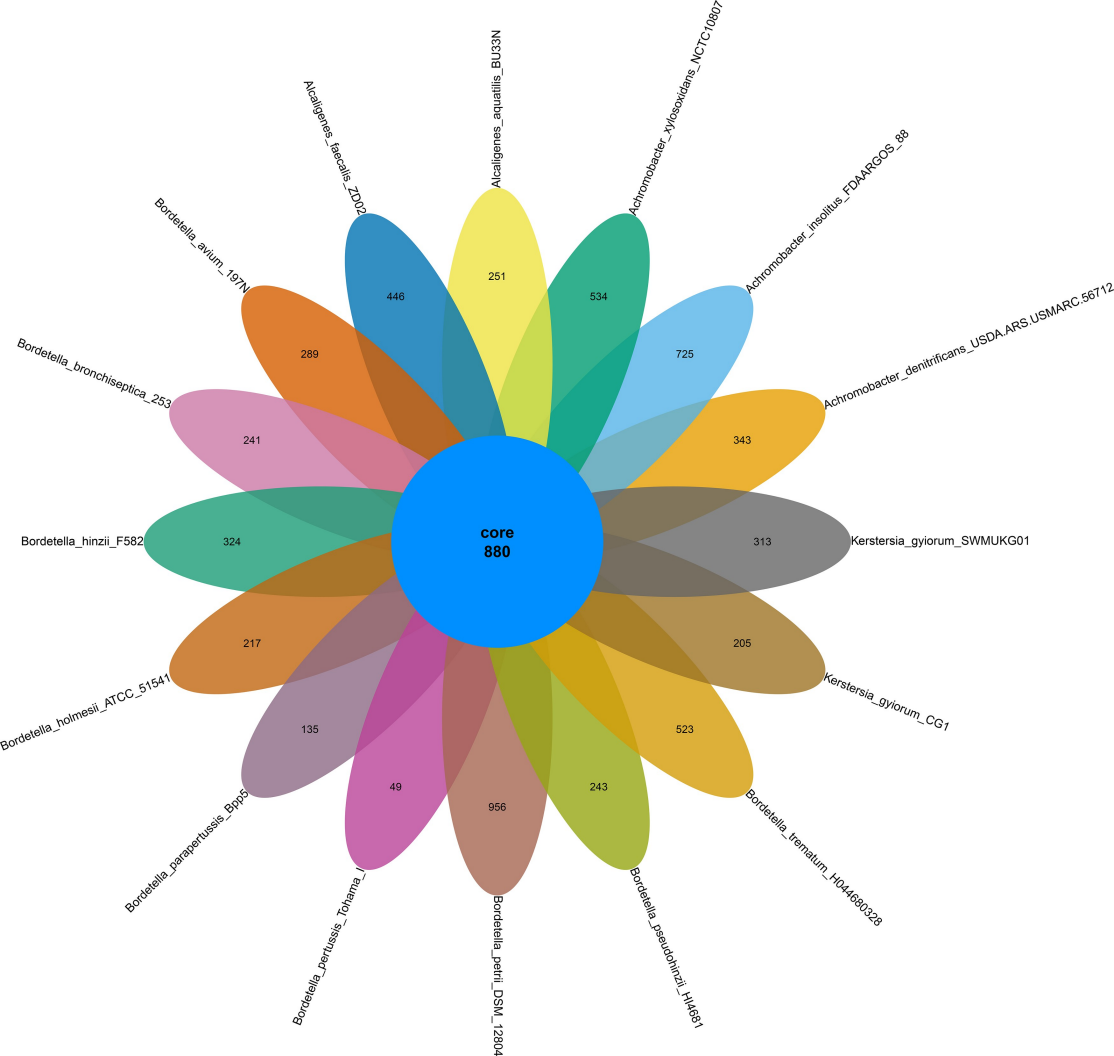
Venn diagram showing the gene conservation among the *K*. *gyiorum* SWMUKG01 and 15 other closely related bacteria. The number in the center of Venn diagram indicates the number of genes shared by all bacteria.

A comparative analysis of the 16 genomes within the family *Alcaligenaceae* was performed to confirm the evolutionary relationship based on the 16S rRNA genes and genome-wide comparisons of orthologous gene pairs. The phylogenetic tree based on the 16S rRNA gene sequences showed that *K*. *gyiorum* SWMUKG01 and CG1 are located on the same node (98.3% similarity) (Fig 2A), both of which are more closely related to *A*. *aquatilis* and *Alcaligenes faecalis*, with 93.53% and 92.9% similarities of *K*. *gyiorum* SWMUKG01 in relative to *A*. *aquatilis* BU33N and *A*. *faecalis* ZD02. Genome-wide comparisons showed that 880 core genes were shared among *K*. *gyiorum* SWMUKG01 and the other 15 closely related species (Fig 3), based on which, the phylogenetic tree was reconstructed and also demonstrated that *K*. *gyiorum* SWMUKG01 and CG1 display closer evolutionary relationship to *A*. *aquatilis* and *A*. *faecalis* than to other species tested (Fig 2B). The strain SWMUKG01 was further confirmed to belong to *K*. *gyiorum* based on the ANI analysis, as it had a 99.1% ANI value with *K*. *gyiorum* CG1, which is obviously above the 95%-96% cut-off usually used to define a bacterial species [31].

### Analysis of virulence factors

326 potential virulence factors were annotated in strain SWMUKG01 genome and these proteins fell into 134 VF terms. Among all these potential virulence factors, proteins involved in flagella and pili production, biosynthesis of lipopolysaccharide and capsule, iron acquisition, secretion and efflux pump systems as well as two-component systems were included (S3 Table). Further studies, e.g. gene knockout studies and animal experiments, were necessary for elucidating the contribution of these virulence factors to pathogenicity of *K*. *gyiorum*. The genomic characteristics of the specific pathogenesis/virulence factors are described in detail below:

#### Flagella biosynthesis

**Fig 4:**
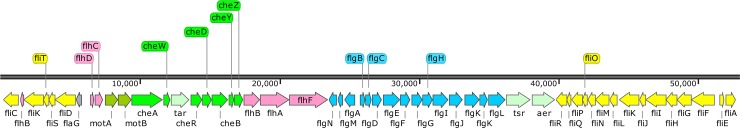
Structure and distribution of the flagellum biosynthetic region (PROKKA_00866–00919) in *K*. *gyiorum* SWMUKG01. The box arrows represent ORFs.

In the family of *Alcaligenaceae*, flagella have been widely identified in the genera of *Alcaligenes*, *Achromobacter* and *Pigmentiphaga* [32, 33]. In *Bordetella*, the flagellar operons of both *B*. *pertussis* and *B*. *parapertussis* are inactivated, leading to inability to make flagella [34]. Of the two species in *Kerstersia*, *K*. *similis* was reported to have no motility due to lacking flagella [4], whereas a full flagellar regulon (a cluster of operons) was identified in the genome of *K*. *gyiorum* SWMUKG01 in this study (Fig 4).

BLASTn searches revealed that the genes of the flagellar regulon in strain SWMUKG01 showed 99% identity with those in *K*. *gyiorum* CG1, which indicates that the flagellar system in *K*. *gyiorum* is highly conserved. The putative flagellar regulon (PROKKA_00866–00919) in SWMUKG01, approximately 52 kb, encodes proteins involved in flagella biosynthesis, export, motor and bacterial chemotaxis (S4 Table). The bacterial chemotaxis system encoded by PROKKA_00877–00884 and PROKKA_00903–00904 shares 71% identity and 72% sequence coverage with those in *Bordetella genomosp*. 8. It is suggested that bacterial chemotaxis operates as important part of a complex network of signaling pathways, by which bacteria adjust and produce an optimal physiological response to an ever-changing environment[35]. Here, we propose that the flagellar system in *K*. *gyiorum* may contribute to its survival in a hostile environment during infection and to the generation of pathogenic responses.

#### Adherence

**Fig 5:**
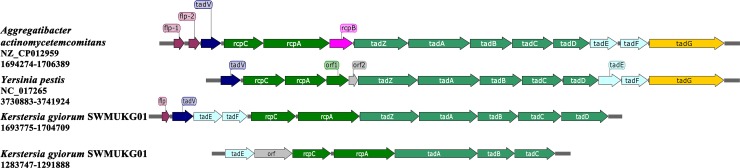
Comparison of the pilus structure in the genomes of *K*. *gyiorum* SWMUKG01, *Aggregatibacter actinomycetemcomitans* and *Y*. *pestis*. The box arrows represent ORFs.

As previously reported, *tad* (tight adherence) genes encode the machinery that is essential for the assembly of adhesive Flp (fimbrial low-molecular-weight protein) pili, which are required for autoaggregation, colonization, biofilm formation and pathogenesis in the genera *Actinobacillus*, *Haemophilus*, *Pseudomonas*, *Yersinia* and perhaps others [36]. Analysis of the genome sequence facilitates the identification of a *tad* gene cluster *tadZABCD* (*PROKKA_01524–01528*) in the strain SWMUKG01 (Fig 5), which is highly conserved in *K*. *gyiorum* showing 96% identity with that in CG1. Similar *tad* loci have also been identified in several Gram-negative pathogens, such as *Pasteurella multocida*, *Yersinia pestis* and *Vibrio cholerae* [36, 37]. Based on sequence comparisons in the family of *Alcaligenaceae*, the *tadABC* genes in *K*. *gyiorum* SWMUKG01 are highly homologous to those in some other members. For instance, the *tadABC* in strain SWMUKG01 showed 78%, 74% and 75% identities comparing to those of *A*. *xylosoxidans* and 78%, 74% and 70% identities with that of *B*. *pertussis*, respectively. Despite low homologies with those from other species, the putative protein encoded by *tadZ* in strain SWMUKG01 was predicted to be involved in localization of pilus biogenesis and TadD potentially contained a tetratricopeptide repeat protein–protein interaction motif, which is required for the assembly of Flp pili [36].

Furthermore, we found that the *flp-tadVEF-rcpCA* gene region was closely linked to the *tad* cluster in SWMUKG01 genome, forming a 11-gene cluster *flp-tadVEF-rcpCA-tadZABCD* (PROKKA_01518–01528) that is predicted to be involved in the biosynthesis and secretion of Flp pili [36] (Table 2). However, *flp* and *tadV* of the cluster were missing in CG1. By a further search in the SWMUKG01 genome, we found another incomplete subset of *tad* operon, *tadE-orf-rcpCA-tadABC* (PROKKA_01139–01145) (Fig 5). Due to the lack of *flp* gene, which encodes the major structural component of Flp pili, this truncated *tad* cluster is likely to be null. It is known that the Flp pili are a distinct clade of type IVb pili [38]. To explore the existence of other types of pili in strain SWMUKG01, we searched all the genes encoding proteins for pilus biosynthesis in the genome in addition to the two *tad* clusters described above. As a result, a few genes, scattered throughout the SWMUKG01 chromosome, were found coding for proteins putatively involved in the type IVa pilus biosynthesis, including *pilZ* (PROKKA_00005), *pilF* (PROKKA_00008), *pilD* (PROKKA_00160) and *pilP* (PROKKA_02422). However, these CDs seem to be incomplete for the type IVa pili production, as the gene coding for the main pilus subunit pilin (PilA), a central component, was not found [38]. Besides, genes encoding potential functional analogues of type 1 or P pili are also absent in the SWMUKG01 genome.

**Table 2:** Genes encoding proteins with a role in adherence of strain SWMUKG01.

CDS no.	Putative Product	Name
PROKKA_01139	TadE-like family protein	*tadE*
PROKKA_01140	hypothetical protein	-
PROKKA_01141	Flp pilus assembly protein CpaB	*rcpC*
PROKKA_01142	Flp pilus assembly protein, secretin CpaC	*rcpA*
PROKKA_01143	pilus assembly protein CpaF	*tadA*
PROKKA_01144	Flp pilus assembly protein TadB	*tadB*
PROKKA_01145	Flp pilus assembly protein TadC	*tadC*
PROKKA_01518	Flp pilus assembly protein, pilin Flp	*flp*
PROKKA_01519	type IV leader peptidase family protein	*tadV*
PROKKA_01520	Flp pilus assembly protein	*tadE*
PROKKA_01521	Flp pilus assembly protein	*tadF*
PROKKA_01522	Flp pilus assembly protein RcpC	*rcpC*
PROKKA_01523	Flp pilus assembly protein, secretin	*rcpA*
PROKKA_01524	Flp pilus assembly protein, ATPase	*tadZ*
PROKKA_01525	pilus assembly protein TadA	*tadA*
PROKKA_01526	Flp pilus assembly protein TadB	*tadB*
PROKKA_01527	Flp pilus assembly protein TadC	*tadC*
PROKKA_01528	Flp pilus assembly protein TadD, contains TPR repeats	*tadD*

#### Biosynthesis of lipopolysaccharides (LPSs)

Surface polysaccharides are extremely diverse and occur in multiple forms in Gram-negative bacteria, such as lipopolysaccharides (LPSs), the essential components of the outer membrane structurally and functionally, and capsular polysaccharides, surface layers or capsules that are associated with the cell [39].

All genes encoding enzymes for biosynthesis of the lipid A and core OS in the SWMUKG01 genome were identified (Table 3), which are highly conserved in *K*. *gyiorum* showing no less than 94% identities with those in CG1. These CDSs are scattered throughout the SWMUKG01 chromosome, just like most Gram-negative bacteria, such as *E*. *coli*, *Neisseria meningitidis*, *Y*. *pestis* and *Pseudomonas aeruginosa*. The majority of genes essential for the synthesis of lipid A (*lpxK*, *lpxD*, *lpxA*, *lpxB*, *lpxC*, *lpxL*, *kdsA*, *kdsC* and *kdsD*) and KDO core oligosaccharide (*waaP*, *waaC*, *waaG*, *waaA*, *waaF*, *waaE*, *lpsB* and *gmhA*) are highly conserved among different species within the family *Alcaligenaceae*. However, *kdsB* in *K*. *gyiorum* SWMUKG01, encoding CMP-2-keto-3-deoxyoctulosonic acid synthetase involved in lipid A synthesis, showed little similarity with that in other *Alcaligenaceae* members, but shared 71% sequence identity in *Pseudomonas monteilii* and *Pseudomonas citronellolis*. *lpsE*, encoding glycosyltransferase in core OS biosynthesis, showed 67% identity (98% sequence coverage) with that in *Stenotrophomonas rhizophila* but only 23% coverage in *B*. *pertussis*. The gene *msbA* (PROKKA_01899) is also identified encoding a putative lipid A export ATP-binding/permease protein (583aa) that is required for the fiipping of lipid A / core moiety of LPS from the cytoplasmic side of the IM to the periplasmic face.

**Table 3:** Genes encoding proteins with a role in lipopolysaccharide metabolism of strain SWMUKG01.

CDS no.	Putative Product	Name
PROKKA_00186	lipopolysaccharide export system permease protein LptG	*lptG*
PROKKA_00221	glycosyltransferases	-
PROKKA_00277	phosphoheptose isomerase	*gmhA*
PROKKA_00294	lipopolysaccharide export system ATP-binding protein LptB	*lptB*
PROKKA_00295	lipopolysaccharide transport periplasmic protein LptA	*lptA*
PROKKA_00296	ABC transporter, LPS-binding protein LptC	*lptC*
PROKKA_00297	3-deoxy-D-manno-octulosonate8-phosphate phosphatase KdsC	*kdsC*
PROKKA_00298	arabinose 5-phosphate isomerase KdsD	*kdsD*
PROKKA_00500	phospho-N-acetylmuramoyl-pentapeptide-transferase	*wecA*
PROKKA_00621	prolipoprotein diacylglyceryl transferase	*lgt*
PROKKA_01031	phospho-2-dehydro-3-deoxyheptonate aldolase, Phe-sensitive	-
PROKKA_01063	lipopolysaccharide core heptosyltransferase RfaQ	*waaC*
PROKKA_01064	lipid A export ATP-binding/permease protein MsbA	*msbA*
PROKKA_01162	ATP-dependent zinc metalloprotease FtsH 2	*ftsH2*
PROKKA_01172	phospho-2-dehydro-3-deoxyheptonate aldolase, Phe-sensitive	-
PROKKA_01290	MobA-like NTP transferase domain protein	-
PROKKA_01291	phosphotransferase enzyme family protein	-
PROKKA_01292	LPS-assembly protein LptD precursor	*lptD*
PROKKA_01350	dTDP-4-dehydrorhamnose 3,5-epimerase	*rfbC*
PROKKA_01351	Glucose-1-phosphate thymidylyltransferase 1	*rfbA*
PROKKA_01352	dTDP-4-dehydrorhamnose reductase	*rfbD*
PROKKA_01353	dTDP-glucose 4,6-dehydratase	*rfbB*
PROKKA_01539	tetraacyldisaccharide 4'-kinase	*lpxK*
PROKKA_01756	UDP-3-O-acylglucosamine N-acyltransferase	*lpxD*
PROKKA_01757	3-hydroxyacyl-[acyl-carrier-protein] dehydratase FabZ	*fabZ*
PROKKA_01758	acyl-[acyl-carrier-protein]-UDP-N-acetylglucosamine O-acyltransferase	*lpxA*
PROKKA_01759	lipid-A-disaccharide synthase	*lpxB*
PROKKA_01899	lipid A export ATP-binding/permease protein MsbA	*msbA*
PROKKA_01912	UDP-glucose 6-dehydrogenase Ugd	*ugd*
PROKKA_02020	UDP-N-acetylgalactosamine-undecaprenyl-phosphate N-acetylgalactosaminephosphotransferase	*wcaJ*
PROKKA_02021	putative glycosyltransferase EpsD	*espD*
PROKKA_02022	glycosyltransferase	*rfaG*
PROKKA_02023	putative O-antigen transporter	*wzx*
PROKKA_02311	3-deoxy-D-manno-octulosonic acid kinase	*waaP*
PROKKA_02386	phosphoglucosamine mutase GlmM	*glmM*
PROKKA_02388	ATP-dependent zinc metalloprotease FtsH	*ftsH*
PROKKA_02665	3-deoxy-manno-octulosonate cytidylyltransferase	*kdsB*
PROKKA_02666	2-dehydro-3-deoxyphosphooctonate aldolase	*kdsA*
PROKKA_02674	D-beta-D-heptose 7-phosphate kinase	*rfaE*
PROKKA_02675	tetratricopeptide repeat protein YciM	*yciM*
PROKKA_02726	LPS-assembly lipoprotein LptE	*lptE*
PROKKA_02751	lipopolysaccharide export system permease protein LptF	*lptF*
PROKKA_02821	lipopolysaccharide export system ATP-binding protein LptB	*lptB*
PROKKA_02830	lipid A deacylase PagL precursor	*pagL*
PROKKA_02933	UDP-3-O-acyl-N-acetylglucosamine deacetylase	*lpxC*
PROKKA_02960	glycosyltransferase	*rfaG*
PROKKA_02961	glycosyltransferase	-
PROKKA_02962	glycosyltransferase	-
PROKKA_02963	putative peptidoglycan biosynthesis protein MurJ	*murJ*
PROKKA_02964	UDP-glucose 4-epimerase	*WcaG*
PROKKA_02965	UDP-N-acetyl-D-glucosamine 6-dehydrogenase	*wecC*
PROKKA_02966	Undecaprenyl-phosphate alpha-N-acetylglucosaminyl 1-phosphate transferase	*wecA*
PROKKA_03199	glycosyltransferase	*mgtA*
PROKKA_03200	hypothetical protein	-
PROKKA_03201	S-adenosylmethionine synthase	-
PROKKA_03202	Lipid A biosynthesis lauroyl acyltransferase, HtrB	*lpxL*
PROKKA_03203	Lipid A biosynthesis lauroyl acyltransferase, HtrB	*lpxL*
PROKKA_03256	3-deoxy-D-manno-octulosonic acid transferase WaaA	*waaA*
PROKKA_03257	lipopolysaccharide heptosyltransferase 1 RfaF	*waaF*
PROKKA_03278	UDP-glucose 4-epimerase	*wcaG*
PROKKA_03378	glutamine—fructose-6-phosphate aminotransferase	*glmS*
PROKKA_03380	O-antigen ligase	*wzy*
PROKKA_03381	putative polysaccharide deacetylase YxkH	*yxkH*
PROKKA_03382	lipopolysaccharide core biosynthesis glycosyltransferase LpsE	*lpsE*
PROKKA_03383	lipopolysaccharide core biosynthesis mannosyltransferase LpsB	*lpsB*
PROKKA_03384	lipopolysaccharide core biosynthesis glycosyltransferase WaaE	*waaE*
PROKKA_03385	predicted xylanase/chitin deacetylase YadE	*yadE*
PROKKA_03386	bifunctional protein GlmU	*glmU*
PROKKA_03520	UDP-glucose 6-dehydrogenase	*ugd*

9 CDSs, showing more than 96% identities with those in CG1, that are likely associated with O-antigen biogenesis were predicted in the SWMUKG01 genome. These CDSs could be classified into three groups: nucleotide sugar synthetases by a potential *rfb* operon (*rfb*CADB, PROKKA_01350–01353), glycosyltransferase genes *wcaG*, *wecC*, and *wecA* (PROKKA_02964–02966) and oligosaccharide repeat unit processing genes *wzy* (PROKKA_03380) and *wzx* (PROKKA_02023). Of these CDSs, only genes encoding the dTDP-glucose 4, 6-dehydratase RfbB and UDP-N-acetyl-D-glucosamine 6-dehydrogenase WecC are conserved across a wide range of species in *Alcaligenaceae* family, such as *B*. *bronchiseptica* and *B*. *pertussis*. It is known that in the Wzy-dependent pathway implicated in the assembly or export of O-antigen, O-unit polymerase Wzy, O-unit flippase Wzx and the O-chain length determinant Wzz are included [40]. However, we could not detect the *wzz* homolog in *K*. *gyiorum* SWMUKG01. Further experimental works are required to verify the O-antigen-processing process.

#### Capsular polysaccharides (CPS)

A cluster of genes coding for proteins involved in CPS biosynthesis and export were identified in the strain SWMUKG01 ranging from PROKKA_01910 to PROKKA_01935 (Table 4). However, several genes from PROKKA_01920 to PROKKA_01928 in this cluster were not detected in CG1, which might be due to the incomplete genome sequence. The G+C content of this gene cluster is much lower (53%) than that of the SWMUKG01 chromosome (62%), which is similar to the O-antigen’s case in many Gram-negative bacteria [41]. In fact, the genetic loci for CPS production in *K*. *gyiorum* SWMUKG01 seem to be allelic to many LPS biosynthetic loci, and multiple enzymes are the same between these pathways, such as flippase Wzx, glycosyltransferase RfaG and UDP-N-acetyl-D-mannosaminuronate dehydrogenase WecC. It is known that Wza, Wzb, and Wzc are translocation proteins that specifically export group I or IV CPS to the outer surface. The identification of *wza*, *wzb*, and *wzc* genes suggested a type of group 1 or IV CPS in *K*. *gyiorum* SWMUKG01. It is reported that the initiating glycosyltransferase is the distinguishing factor between group I and IV CPS biosynthesis pathways, with WbaP catalyzing group I and WecA for group IV CPS biosynthesis. By searching in the SWMUKG01 genome, we could not identify a *wbaP* homologue, but two *wecA* (PROKKA_02966 and PROKKA_00500) were identified though it locates outside the CPS gene cluster. We proposed that the strain SWMUKG01 appears to have a group IV CPS. As group IV CPS biosynthesis is Wzy-dependent, the O-antigen polymerase Wzy appears to participate in both CPS and LPS biosynthesis in *K*. *gyiorum* SWMUKG01, asin *Vibrio vulnificus* [42].

**Table 4:** Genes encoding proteins with a role in capsule polysaccharide biosynthesis of strain SWMUKG01.

CDS no.	Putative Product	Name
PROKKA_01911	glucose-6-phosphate isomerase	*pgi*
PROKKA_01912	UDP-glucose 6-dehydrogenase TuaD	*ugd*
PROKKA_01913	UTP—glucose-1-phosphate uridylyltransferase	*galU*
PROKKA_01914	probable low molecular weight protein-tyrosine-phosphatase EpsP	*wzb*
PROKKA_01918	putative tyrosine-protein kinase EpsB	*wzc*
PROKKA_01919	putative polysaccharide export protein Wza	*wza*
PROKKA_01920	glycosyltransferase	*rfaG*
PROKKA_01924	glycosyltransferase	-
PROKKA_01926	D-inositol-3-phosphate glycosyltransferase	-
PROKKA_01927	polysaccharide biosynthesis protein RfbX	*wzx*
PROKKA_01928	uncharacterized glycosyltransferase YpjH	*ypjH*
PROKKA_01929	UDP-N-acetyl-D-glucosamine 6-dehydrogenase	*wecC*
PROKKA_01931	L-glutamine:2-deoxy-scyllo-inosose aminotransferase	*wecE*
PROKKA_01932	capsular polysaccharide biosynthesis protein CapD	*capD*
PROKKA_01933	probable polysaccharide biosynthesis protein EpsC	*epsC*
PROKKA_01934	putative sugar transferase EpsL	*epsL*
PROKKA_01935	Putative acetyltransferase EpsM	*epsM*

Analysis on the SWMUKG01 genome sequence identified three genomic islands (GIs) (S5 Table), which were defined by obviously different GC contents in comparison to the average of the genome and associated with the presence of insertion sequences, integrases and transposases. We found that the CPS gene cluster is mainly composed of a specific genomic island GI-III (PROKKA_01918–01931) and several genes on both sides. The GI-III is a 16, 772 bp island with a GC content of 49% and codes for 14 proteins, including those for the biosynthesis and transport of capsular polysaccharides in addition to an IS2 transposase TnpB. The identification of GI-III suggested that these capsular polysaccharide encoding genes might have evolved from a different organism by horizontal gene transfer. Besides, the GI-III presents some characteristics of pathogenicity islands for the presence of some putative virulence related genes (PROKKA_01918, 01919, 01920, 01929 and 01931).

#### Secretion systems

The SWMUKG01 genome harbors complete sets of genes coding for proteins that constitute the general secretion (Sec) and two-arginine (Tat) pathways (Table 5), which are essential for export of proteins into the periplasm (Gram-negative bacteria) or plasma membrane (Gram-positive bacteria) [43]. Three sets of closely linked genes encoding the putative T1SS, namely *prsD*1-*prsE*1-*tolC*1 (PROKKA_00929–00931), *tolC*2-*prsD*2-*prsE*2 (PROKKA_01512–01514) and *prsE*3-*prsD*3-*tolC*3 (PROKKA_02054–02056), were identified on this genome (Table 5). The identities among these three T1SSs were 33.5 ± 4.9%, suggesting a structural independence between each other. A further blast showed that *prsD*1-*prsE*1-*tolC*1 of the strain SWMUKG01 had 83% identity and 93% coverage with that from *Comamonas Kerstersii*, and *prsE*3-*prsD*3-*tolC*3 obtained 70% identity and 92% coverage with that from *Variovorax Boronicumulans*, but *tolC*2-*prsD*2-*prsE*2 had no significant homologues in the GenBank.

**Table 5:** Genes encoding secretion systems in strain SWMUKG01.

CDS no.	Putative Product	Name
PROKKA_00248	protein-export protein SecB	*secB*
PROKKA_01637	protein-export membrane protein SecG	*secG*
PROKKA_02930	protein translocase subunit SecA	*secA*
PROKKA_02951	protein translocase subunit SecD	*secD*
PROKKA_02952	protein-export membrane protein SecF	*secF*
PROKKA_03340	protein translocase subunit SecY	*secY*
PROKKA_03503	protein translocase subunit SecE	*secE*
PROKKA_03421	Sec-independent protein translocase protein TatA	*tatA*
PROKKA_03422	Sec-independent protein translocase protein TatB	*tatB*
PROKKA_03423	Sec-independent protein translocase protein TatC	*tatC*
PROKKA_00929	type I secretion system ATP-binding protein PrsD	*prsD1*
PROKKA_00930	type I secretion system membrane fusion protein PrsE	*PrsE1*
PROKKA_00931	outer membrane protein TolC precursor	*tolC1*
PROKKA_01512	outer membrane protein TolC precursor	*tolC2*
PROKKA_01513	alpha-hemolysin translocation ATP-binding protein HlyB	*prsD2*
PROKKA_01514	type I secretion system membrane fusion protein PrsE	*PrsE2*
PROKKA_02054	type I secretion system membrane fusion protein PrsE	*prsE3*
PROKKA_02055	alpha-hemolysin translocation ATP-binding protein HlyB	*PrsD3*
PROKKA_02056	outer membrane protein TolC precursor	*tolC3*
PROKKA_02423	type II secretion system protein G, pseudopilin	*gspG*
PROKKA_02424	type II secretion system protein H, pseudopilin	*gspH*
PROKKA_02425	type II secretion system protein I, pseudopilin	*gspI*
PROKKA_02426	type II secretion system protein J, pseudopilin	*gspJ*
PROKKA_02427	type II secretion system protein K, pseudopilin	*gspK*
PROKKA_02428	inner membrane platform protein GspL	*gspL*
PROKKA_02429	inner membrane platform protein GspM	*gspM*
PROKKA_02430	tRNA-Arg(tcg)	-
PROKKA_02431	type II secretion system protein D, secretin	*gspD*
PROKKA_02432	type II secretion system protein E, ATPase	*gspE*
PROKKA_02433	type II secretion system protein F	*gspF*
PROKKA_00412	type IV secretion system protein VirD4, ATPases	*virD4*
PROKKA_00413	CopG family transcriptional regulator	*copG*
PROKKA_00414	type IV secretion system protein VirB11, ATPases	*virB11*
PROKKA_00415	type IV secretory pathway, VirB2 components (pilins)	*virB2*
PROKKA_00416	type IV secretory pathway, VirB3 components	*virB3*
PROKKA_00417	type IV secretion system protein VirB4, ATPases	*virB4*
PROKKA_00418	conjugative transfer protein TrbJ	*virB5*
PROKKA_00419	hypothetical protein	-
PROKKA_00420	type IV secretory pathway, VirB6 components	*virB6*
PROKKA_00421	type IV secretory pathway, VirB8 components	*virB8*
PROKKA_00422	type IV secretory pathway, VirB9 components	*virB9*
PROKKA_00423	type IV secretory pathway, VirB10 components	*virB10*
PROKKA_02491	type IV secretory pathway, VirB10 components	*virB10*
PROKKA_02492	type IV secretory pathway, VirB9 components	*virB9*
PROKKA_02493	type IV secretory pathway, VirB8 components	*virB8*
PROKKA_02494	type IV secretory pathway, VirB6 components	*virB6*
PROKKA_02495	conjugative transfer protein TrbJ	*virB5*
PROKKA_02496	type IV secretion system protein VirB4, ATPases	*virB4*
PROKKA_02497	type IV secretion system protein VirB4, ATPases	*virB4*
PROKKA_02498	type IV secretory pathway, VirB3 components	*virB3*
PROKKA_02499	type IV secretory pathway, VirB2 components (pilins)	*virB2*
PROKKA_02500	type IV secretion system protein VirB11, ATPases	*virB11*
PROKKA_02501	CopG family transcriptional regulator	*copG*
PROKKA_02502	type IV secretion system protein VirD4, ATPases	*virD4*

*K*. *gyiorum* harbors a putative *gsp* operon encoding proteins putatively involved in the biosynthesis of T2SS (Table 5). This *gsp* operon, consisting of *gspGHIJKLMDEF*, is located between PROKKA_02423 and PROKKA_02433, with an unexpected tRNA encoding gene (PROKKA_02430) inserted into the operon. It seems that GspC and GspO proteins were lacking in the strain SWMUKG01, for that a typical T2SS apparatus bears 12 core components (T2SS CDEFGHIJKLMO) [44]. These gene absences do not necessarily mean that this bacterium lacks a functioning system, because some T2SS proteins, especially C, could have been missed as a result of them being the least conserved among core constituents [44]. More work is needed to determine whether the T2SS homologs in this strain are expressed correctly and encode a functional secretion system.

**Fig 6:**
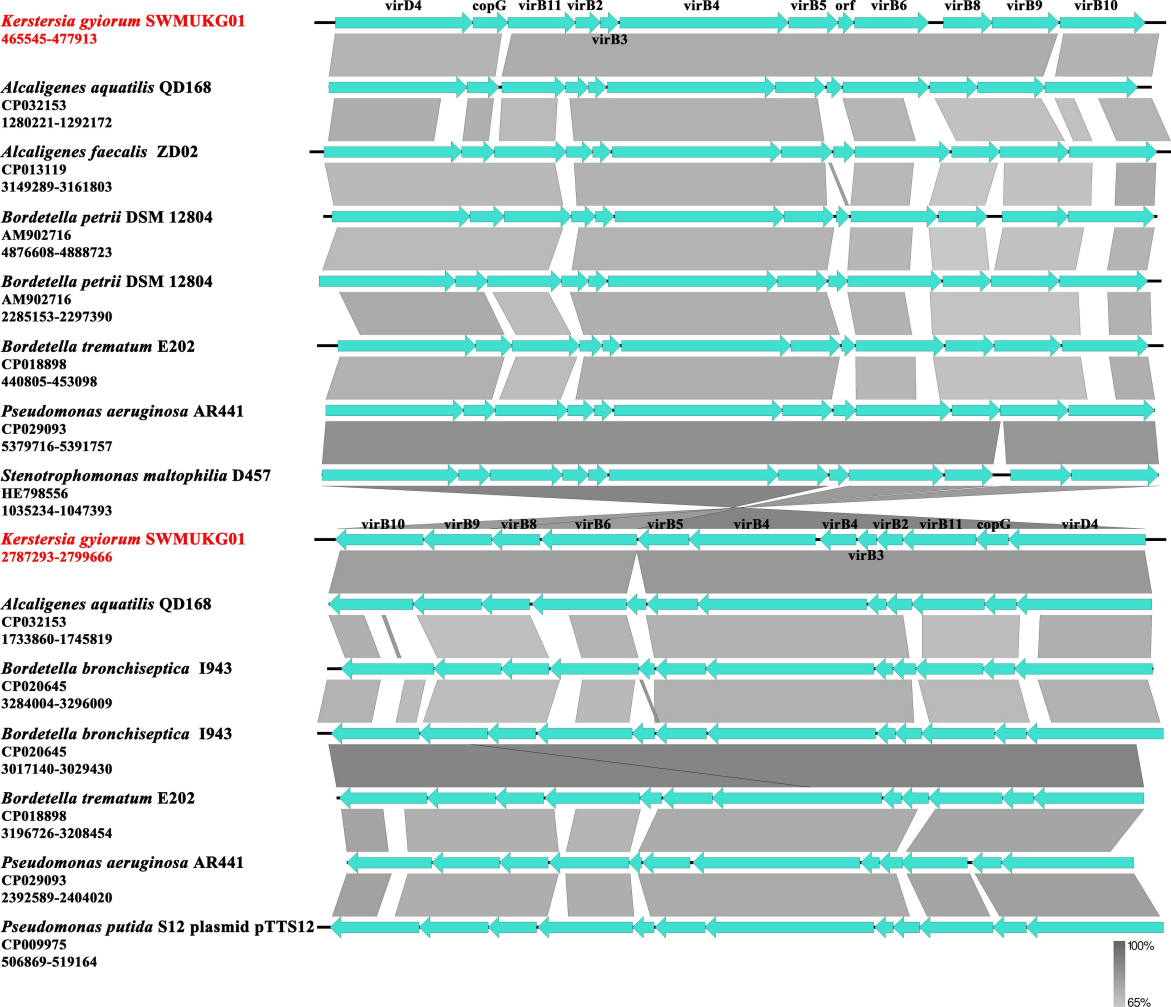
Structure and distribution of T4SS in *K*. *gyiorum* SWMUKG01 and other related bacteria. The box arrows represent ORFs. Similar regions are indicated with the degree of nucleotide identity being shown in gray scales.

An interesting finding on the SWMUKG01 genome was the presence of two sets of genes encoding proteins homologous to the conjugation paradigm VirB/D T4SS, which we designated the *vir1* (PROKKA_00412–00423) and *vir2* (PROKKA_02490–02502) locus (Table 5). The nucleotide sequence identity between these two sets of genes was 77%, indicating that they were very likely to be duplicated copies. Compared with that in *vir2* locus, almost the same gene context was contained in *vir1*, except that double *virB4* were found in the former but a single in the latter (Fig 6). Both *vir1* and *vir2* showed highest identity (75% and 73%) with those of *A*. *aquatilis*, and other homologous T4SS were also found in *Bordetella petrii*, *Bordetella trematum*, *B*. *bronchiseptica* and *P*. *aeruginosa* etc (Fig 6). It was unexpected to identify a *copG* gene between *virB11* and *virD4* in both *vir* loci of SWMUKG01. Similar gene arrangement occurs in a conserved fashion in many other bacteria. It is known that CopG is a transcriptional repressor that control the plasmid copy number [45]. And, we found a homologous T4SS encoded by a plasmid pTTS12 in *Pseudomonas putida*, also with a *copG* gene between *virB11* and *virD4*. Thus, we proposed that the T4SS encoding system on the SWMUKG01 chromosome is most likely to originate from a plasmid by horizontal transfer. Besides, in comparison with the VirB/VirD4 system of *Agrobacterium tumefaciens* [46], an archetypal T4SS that is encoded by the *vir* gene cluster composed of 12 components, *virB1*–*virB11* and *virD4*, SWMUKG01 lacks *virB7* in both *vir* loci. However, on the SWMUKG01 genome, a hypothetical protein (PROKKA_00419) is placed between *virB5* and *virB6*. Similar gene arrangements also occur in other homologous T4SSs. This suggested that the hypothetical protein may play similar roles as the VirB7 does. If this is true and both *vir1* and *vir2* are functional, it would be interesting to learn these two T4SS systems coexist in SWMUKG01. Furthermore, the presence of secretion systems in CG1 was investigated; the results showed that all the secretion systems discussed above could be detected except the *vir1* locus of T4SS.

#### Iron-uptake systems

Three broad categories of iron-uptake systems have been currently identified: systems for the utilization of ferric iron (Fe^3+^), ferrous iron (Fe^2+^) and heme-bound iron. By searching the genome, we found that the SWMUKG01 harbors genes encoding proteins for all three categories of iron-uptake systems.

Approximately 88 genes (2.5%) of the SWMUKG01 genome are involved in iron uptake (Table 6), all of which, except the genes from PROKKA_00721 to PROKKA_00723, could also be detected in CG1. Among them, 16 genes encoding potential outer membrane receptors for ferric siderophores, of which seven are involved in binding ferric enterobactin, four binding ferric dicitrate, two binding ferric-pseudobactin, one binding ferripyoverdine, one binding ferrichrome and one binding ferrichrysobactin. In addition, 8 CDSs for putative TonB-dependent receptor precursors were also identified. Interestingly, all of them are located in a potential operon with *fecI* and *fecR*, regulatory genes of the iron dicitrate transport systems, which may indicate a functional relevance between the putative receptor precursors and FecIR for iron uptake. Although the SWMUKG01 possesses multiple outer membrane receptors, each of which provides the bacterium with specificity for different siderophores, it only contains three binding-protein-dependent ABC systems: FepCBDG (PROKKA_00824–00827) for the transport of ferric catechols, FecBCDE (PROKKA_02160–02163) for ferric citrate and FhuBCD (PROKKA_02518–02521) for ferric hydroxamates. In addition, a putative *fbp* operon (PROKKA_01345–01347) encoding the FbpABC system was found on SWMUKG01 genome. It was previously shown that FbpABC is likely to be a ferric iron transporter that is involved in the translocation of iron delivered by transferrin and lactoferrin, across the cytosolic membrane [47]. Interestingly, no gene encoding putative transferrin or lactoferrin receptor has been found in this organism, leading to the hypothesis that the FbpABC system functions in the utilization of some siderophores as iron source in SWMUKG01 [48].

**Table 6:** Genes encoding iron uptake systems in strain SWMUKG01.

CDS no.		Putative Product	Name
PROKKA_00020		putative TonB-dependent receptor precursor	*tonB*
PROKKA_00021		*fec* operon regulator FecR	*fecR*
PROKKA_00022		putative RNA polymerase sigma factor FecI	*fecI*
PROKKA_00473		ferric enterobactin receptor precursor FepA	*fepA*
PROKKA_00474		putative TonB-dependent receptor precursor	*tonB*
PROKKA_00475		putative TonB-dependent receptor precursor	*tonB*
PROKKA_00476		*fec* operon regulator FecR	*fecR*
PROKKA_00477		putative RNA polymerase sigma factor FecI	*fecI*
PROKKA_00585		ferrous iron permease EfeU	*efeU*
PROKKA_00586		deferrochelatase/peroxidase EfeB precursor	*efeB*
PROKKA_00587		iron uptake system component EfeO precursor	*efeO*
PROKKA_00721		putative RNA polymerase sigma factor FecI	*fecI*
PROKKA_00722		*fec* operon regulator FecR	*fecR*
PROKKA_00723		Fe (3+) dicitrate transport protein FecA precursor	*fecA*
PROKKA_00724		PKD domain protein	-
PROKKA_00725		putative RNA polymerase sigma factor FecI	*fecI*
PROKKA_00726		*fec* operon regulator FecR	*fecR*
PROKKA_00727		putative TonB-dependent receptor precursor	*tonB*
PROKKA_00746		ferric-pseudobactin BN7/BN8 receptor precursor	*pupB*
PROKKA_00824		ferric enterobactin transport system permease protein FepG	*fepG*
PROKKA_00825		ABC-type Fe3+-siderophore transport system, permease component FepD	*fepD*
PROKKA_00826		ferrienterobactin-binding periplasmic protein precursor, FepB	*fepB*
PROKKA_00827		putative siderophore transport system ATP-binding protein FepC	*fepC*
PROKKA_00830		Fe (3+) dicitrate transport protein FecA precursor	*fecA*
PROKKA_00863		ferrichrysobactin receptor	-
PROKKA_00923		iron import ATP-binding/permease protein IrtA	*irtA*
PROKKA_01049		Fe (3+) dicitrate transport protein FecA precursor	*fecA*
PROKKA_01050		*fec* operon regulator FecR	*fecR*
PROKKA_01051		putative RNA polymerase sigma factor FecI	*fecI*
PROKKA_01281		putative TonB-dependent receptor precursor	*tonB*
PROKKA_01282		putative RNA polymerase sigma factor FecI	*fecI*
PROKKA_01283		*fec* operon regulator FecR	*fecR*
PROKKA_01345		Fe (3+) ions import ATP-binding protein FbpC	*fbpC*
PROKKA_01346		Fe (3+)-transport system permease protein FbpB	*fbpB*
PROKKA_01347		iron ABC transporter substrate-binding protein FbpA	*fbpA*
PROKKA_01738		TonB-dependent receptor for ferrienterochelin and colicins	*fepA*
PROKKA_01769		TonB-dependent receptor for ferrienterochelin and colicins	*fepA*
PROKKA_01870		biopolymer transport protein ExbD	*exbD*
PROKKA_01871		biopolymer transport protein ExbB	*exbB*
PROKKA_01872		periplasmic protein TonB	*tonB*
PROKKA_02160		iron ABC transporter substrate-binding protein, periplasmic component	*fecB*
PROKKA_02161		iron ABC transporter permease protein FecC	*fecC*
PROKKA_02162		iron ABC transporter permease protein FecD	*fecD*
PROKKA_02163		iron (III) dicitrate transport ATP-binding protein FecE	*fecE*
PROKKA_02295		fec operon regulator FecR	*fecR*
PROKKA_02296		putative RNA polymerase sigma factor FecI	*fecI*
PROKKA_02297		ferric-pseudobactin BN7/BN8 receptor precursor	*pupB*
PROKKA_02405		putative TonB-dependent receptor precursor	*tonB*
PROKKA_02406		fec operon regulator FecR	*fecR*
PROKKA_02407		putative RNA polymerase sigma factor FecI	*fecI*
PROKKA_02517		ferrichrome receptor FhuA precursor	*fhuA*
PROKKA_02518		putative ABC transporter solute-binding protein, FhuD	*fhuD*
PROKKA_02519		iron (3+)-hydroxamate import ATP-binding protein FhuC	*fhuC*
PROKKA_02520		iron ABC transporter permease	*fhuB*
PROKKA_02521		iron ABC transporter permease	*fhuB*
PROKKA_02616		*fec* operon regulator FecR	*fecR*
PROKKA_02617		putative RNA polymerase sigma factor FecI	*fecI*
PROKKA_02621		receptor for ferrienterochelin and colicins	*fepA*
PROKKA_02631		putative RNA polymerase sigma factor FecI	*fecI*
PROKKA_02632		*fec* operon regulator FecR	*fecR*
PROKKA_02633		TonB-dependent heme receptor A precursor	*fepA*
PROKKA_02635		ferric uptake regulation protein	*fur*
PROKKA_02755		putative TonB-dependent receptor precursor	*tonB*
PROKKA_02756		*fec* operon regulator FecR	*fecR*
PROKKA_02757		putative RNA polymerase sigma factor FecI	*fecI*
PROKKA_02782		PKD domain protein	-
PROKKA_02783		Fe (3+) dicitrate transport protein FecA precursor	*fecA*
PROKKA_02784		*fec* operon regulator FecR	*fecR*
PROKKA_02785		putative RNA polymerase sigma factor FecI	*fecI*
PROKKA_02796		ferric enterobactin receptor precursor	*fepA*
PROKKA_03003		colicin I receptor precursor	-
PROKKA_03042		ferric enterobactin receptor precursor	*fepA*
PROKKA_03097		*fec* operon regulator FecR	*fecR*
PROKKA_03098		putative RNA polymerase sigma factor FecI	*fecI*
PROKKA_03175		putative RNA polymerase sigma factor FecI	*fecI*
PROKKA_03176		*fec* operon regulator FecR	*fecR*
PROKKA_03177		ferripyoverdine receptor precursor	*fpvA*
PROKKA_03474		iron siderophore receptor protein	*fecA*
PROKKA_03475		*fec* operon regulator FecR	*fecR*
PROKKA_03476		putative RNA polymerase sigma factor FecI	*fecI*
**Heme**			
PROKKA_01191	heme-binding protein A precursor		*hbpA*
PROKKA_01284	heme/hemopexin utilization protein C precursor		*hxuC*
PROKKA_01313	hemin receptor precursor		*hemR*
PROKKA_02939	hemin import ATP-binding protein HmuV		*hmuV*
PROKKA_02940	hemin transport system permease protein HmuU		*hmuU*
PROKKA_02941	hemin-binding periplasmic protein HmuT precursor		*hmuT*
PROKKA_03318	hemin receptor precursor		*tdhA*
PROKKA_03474	hemin receptor precursor		*hmuR*

In *K*. *gyiorum* SWMUKG01, five genes encoding CDSs homologous to heme-binding receptors were identified (Table 6). These receptors include one periplasmic heme-binding protein, one hemopexin utilization protein C and three TonB-dependent hemin receptors on the outer membrane, which are likely to be involved in the import of heme by binding it directly or by recognizing its carrier. In addition, orthologs of *hmu*VUT were found in SWMUKG01, which encode an ABC-type heme uptake system, comprising a periplasmic heme-binding protein HmuT, a permease HmuU and an ATPase HmuV. It is likely that the HmuVUT system is the main participant in delivering the heme to the cytosol, as it is the only potential heme transport system identified in SWMUKG01.

It is demonstrated that Fe^2+^ is the dominant form of the element under anaerobic and/or acidic conditions [49]. Only one Fe^2+^-uptake system, EfeUOB, has been identified in SWMUKG01 (Table 6), which is demonstrated to be involved in the uptake of ferrous iron in several bacteria [47]. The *efe* operon (PROKKA_00585–00587) in this bacterium encodes a ferrous iron permease EfeU and two periplasmic proteins EfeB and EfeO in sequential order, with a slight difference in gene order comparing with previous studies [50]. In Gram-negative bacteria, the inner-membrane anchored TonB/ExbB/ExbD complex provides the energy required to transport the associated cargo across the outer-membrane [51]. A set of genes encoding the TonB system, *tonB-exbB-exbD* (PROKKA_01872–01870), were identified in SWMUKG01 genome. In some bacteria, such as *V*. *cholerae* [52] and *P*. *aeruginosa* [53], there is more than one TonB-ExbB-ExbD system, while in SWMUKG01 only one exists. This indicated that the TonB system might be shared by different iron uptake systems and heme transport pathways of SWMUKG01.

#### Two-component signal transduction systems (TCSs)

In *K*. *gyiorum* SWMUKG01, a total of 23 open reading frames were identified as putative RRs, 19 of which are adjacent to genes encoding probable HKs, forming 21 HK/RR pairs, all of which could be identified in CG1 but the gene PROKKA_00858. These histidine kinase and response regulator proteins could be categorized into five groups (Table 7): ten pairs and two single RRs belong to the OmpR subfamily, four pairs fall into the FixJ subfamily, three pairs and a single RR are grouped in the CitB subfamily, one pair is in the NtrC subfamily and the remaining two RRs are members of the CheY subfamily [54]. These TCSs are potentially implicated in regulating several aspects of key processes, such as osmoregulation (EnvZ/OmpR), chemotaxis (CheA/CheY), nitrogen metabolism (NtrY/NtrX), oxygen sensing (AcrA/AcrB) and perhaps pathogenicity mechanisms (QseC/QseB) [55]. Overall, a large number of genes encoding putative TCSs appear to make *K*. *gyiorum* well-equipped to respond to and survive environmental changes during the infection cycle.

**Table 7:** Genes encoding two-component signal transduction systems in strain SWMUKG01.

CDS no.	Putative Product	Name
**OmpR subfamily**		
PROKKA_00218	signal transduction histidine-protein kinase BaeS	*baeS*
PROKKA_00219	transcriptional regulatory protein BaeR	*baeR*
PROKKA_00355	transcriptional activator protein CzcR	*czcR*
PROKKA_00356	sensor protein CzcS precursor	*czcS*
PROKKA_00602	osmolarity sensor protein EnvZ	*envZ*
PROKKA_00603	transcriptional regulatory protein OmpR	*ompR*
PROKKA_00671	sensor protein BasS	*basS*
PROKKA_00672	transcriptional regulatory protein QseB	*qseB*
PROKKA_00856	KDP operon transcriptional regulatory protein KdpE	*kdpE*
PROKKA_00857	hypothetical protein	-
PROKKA_00858	sensor histidine kinase LiaS	*liaS*
PROKKA_01868	sensor protein QseC	*qseC*
PROKKA_01869	transcriptional regulatory protein QseB	*qseB*
PROKKA_02258	signal transduction histidine-protein kinase BaeS	*baeS*
PROKKA_02259	transcriptional regulatory protein BaeR	*baeR*
PROKKA_02453	signal transduction histidine-protein kinase BaeS	*baeS*
PROKKA_02454	transcriptional regulatory protein BaeR	*baeR*
PROKKA_02864	osmolarity sensor protein EnvZ	*envZ*
PROKKA_02863	transcriptional regulatory protein OmpR	*ompR*
PROKKA_02989	response regulators AcrA	*acrA*
PROKKA_02990	aerobic respiration control sensor protein ArcB	*acrB*
PROKKA_01215	sensory transduction protein regX3	*regX3*
PROKKA_00510	transcriptional regulatory protein QseB	*qseB*
**FixJ subfamily**		
PROKKA_00712	sensor histidine kinase NodV	*nodV*
PROKKA_00713	response regulator protein NodW	*nodW*
PROKKA_01693	transcriptional regulatory protein FixJ	*fixJ*
PROKKA_01694	sensor protein FixL	*fixL*
PROKKA_01699	C4-dicarboxylate transport sensor protein DctS	*dctS*
PROKKA_01700	C4-dicarboxylate transport transcriptional regulatory protein DctR	*dctR*
PROKKA_01798	sensor protein FixL	*fixL*
PROKKA_01799	transcriptional regulatory protein FixJ	*fixJ*
**CitB subfamily**		
PROKKA_00858	sensor histidine kinase LiaS	*liaS*
PROKKA_00859	transcriptional regulatory protein DegU	*degU*
PROKKA_02009	oxygen sensor histidine kinase NreB	*nreB*
PROKKA_02010	oxygen regulatory protein NreC	*nrec*
PROKKA_02991	transcriptional regulatory protein RcsB	*rcsB*
PROKKA_02992	sensor histidine kinase RcsC	*rcsC*
PROKKA_00362	glycerol metabolism activator AgmR	*agmR*
**NtrC subfamily**		
PROKKA_00239	signal transduction histidine kinase NtrY	*ntrY*
PROKKA_00240	nitrogen assimilation regulatory protein NtrX	*ntrX*
**CheY subfamily**		
PROKKA_00877	chemotaxis protein histidine kinase CheA	*cheA*
PROKKA_00882	chemotaxis response regulator CheB	*cheB*
PROKKA_00883	chemotaxis protein CheY	*cheY*

#### Antibiotic resistant genes and multidrug efflux pumps

Clinical reports showed that some of *K*. *gyiorum* isolates were resistant to ciprofloxacin [2, 8], colistin [10], cefepime and ceftazidime [7], which suggested potential drug resistance genes or efflux pumps. *K*. *gyiorum* SWMUKG01 showed resistant to ciprofloxacin (>2 μg/ml) and cefuroxime (>16 μg/ml). Our genomic analysis showed that 48 (1.39%) out of 3441 potential CDSs were identified in CARD database (S6 Table). Among them, potential resistance genes encoding proteins against fluoroquinolone (*gyrB*, *gyrA*), sulfonamide, rifampicin, and fosfomycin were included, as well as some other multidrug resistance proteins. However, no plasmid was detected in the strain SWMUKG01, thus plasmid-carried drug resistance genes are not part of drug resistance for this pathogen.

By searching in the SWMUKG01 genome, we found a total of 10 sets of genes encoding multidrug efflux pump systems (Table 8), among which only two genes PROKKA_00804 and PROKKA_01650 were discovered to be truncated. In these pumps, RND (resistance-nodulation-division) family transporters are most commonly found in SWMUKG01, such as, AcrAB-OprM, MexAB-OprM and BepEF-TtgF. MFS (major facilitator superfamily) (e.g., EmrAB-OprM) and ABC (ATP-binding cassette) (e.g., MacAB-TtgC) efflux pumps are also included. These efflux pumps were reported to be responsible for the direct extrusion of many kinds of drugs, such as aminoglycosides, β-lactams, fluoroquinolones, macrolides and chloramphenicol from the cell [56]. Given the presence of various efflux pumps in SWMUKG01, the risk of efflux-mediated multidrug resistance is a real possibility in this organism.

**Table 8:** Genes encoding multidrug efflux pumps in strain SWMUKG01.

CDS no.	Putative Product	Name
PROKKA_00059	multidrug resistance protein AcrA precursor	*acrA*
PROKKA_00060	efflux pump membrane transporter AcrB	*acrB*
PROKKA_00061	outer membrane protein OprM precursor	*tolC*
PROKKA_00163	efflux pump outer membrane protein TtgI precursor	*ttgI*
PROKKA_00164	multidrug resistance protein MdtC	*mdtC*
PROKKA_00165	multidrug resistance protein MdtB	*mdtB*
PROKKA_00166	multidrug resistance protein MdtA precursor	*mdtA*
PROKKA_00754	multidrug resistance protein MdtC	*mdtC*
PROKKA_00755	macrolide export protein MacA	*macA*
PROKKA_00804	outer membrane protein OprM precursor	*oprM*
PROKKA_00805	p-hydroxybenzoic acid efflux pump subunit AaeA	*aaeA*
PROKKA_00806	protein AaeX	*aaeX*
PROKKA_00807	p-hydroxybenzoic acid efflux pump subunit AaeB	*aaeB*
PROKKA_01650	efflux pump outer membrane protein TtgF precursor	*ttgF*
PROKKA_01651	efflux pump membrane transporter BepE	*bepE*
PROKKA_01652	efflux pump periplasmic linker BepF	*bepF*
PROKKA_02027	multidrug resistance protein MdtA	*mdtA*
PROKKA_02028	multidrug ABC transporter ATP-binding protein YbhF	*ybhF*
PROKKA_02029	Inner membrane transport permease YhhJ	*yhhJ*
PROKKA_02218	multidrug export protein EmrB	*emrB*
PROKKA_02219	multidrug export protein EmrA	*emrA*
PROKKA_02352	multidrug resistance protein MexA precursor	*mexA*
PROKKA_02353	multidrug resistance protein MexB	*mexB*
PROKKA_02354	outer membrane protein OprM precursor	*oprM*
PROKKA_02372	outer membrane protein OprM precursor	*oprM*
PROKKA_02373	multidrug export protein AcrF	*acrF*
PROKKA_02374	multidrug efflux pump subunit AcrA precursor	*acrA*
PROKKA_02456	macrolide export protein MacA	*macA*
PROKKA_02457	macrolide export ATP-binding/permease protein MacB	*macB*
PROKKA_02458	putative efflux pump outer membrane protein TtgC precursor	*ttgC*
PROKKA_03460	multidrug resistance protein MdtN	*mdtN*
PROKKA_03461	multidrug resistance protein MdtO	*mdtO*
PROKKA_03462	efflux pump outer membrane protein TtgC precursor	*ttgC*

## Conclusions

In conclusion, the complete genome of *K*. *gyiorum* strain SWMUKG01, the first clinical isolate from southwest China, was sequenced in our present study. The length of the genome is about 3.9 million bps with genomic GC content of 62%. Genomic and phylogenetic comparisons indicated that *K*. *gyiorum*, *A*. *aquatilis* and *A*. *faecalis* may derive from a recent common ancestor. A total of 3441 CDSs were annotated, of which 326 potential virulence factors were predicted by VFDB database. Genes and operons related to bacterial surface polysaccharides, flagella, pili, iron acquisition systems, secretion systems, and TCSs as well as efflux pumps were analyzed at the genomic level and compared with those from other pathogens, which underlined the genetic basis of the pathogenesis and virulence of *K*. *gyiorum*. This work allows the identification of a new bacterial species at the genetic level and provides a foundation for future research into the mechanisms of pathogenesis of *K*. *gyiorum*.

## Supporting information

S1 Fig Functional categorization of the *K*. *gyiorum* SWMUKG01 genome based on COG database. (TIF)

S1 Table Functional categorization of the *K*. *gyiorum* SWMUKG01 genome based on COG database. (XLSX)

S2 Table Classification of genes of *K*. *gyiorum* SWMUKG01 by COG codes. (XLSX)

S3 Table Genes encoding proteins with a putative role in virulence of strain SWMUKG01. (XLSX)

S4 Table Genes encoding proteins with a putative role in flagella biosynthesis of strain SWMUKG01. (XLSX)

S5 Table Putative genomic islands in strain SWMUKG01. (XLSX)

S6 Table Putative antibiotic resistant genes in strain SWMUKG01. (XLSX)
