# Supplementary figures and images for: Genomic characterization of Kerstersia gyiorum SWMUKG01, an isolate from a patient with respiratory infection in China
Source: PLoS One. 2019 Apr 12;14(4):e0214686. doi: 10.1371/journal.pone.0214686 (PMC6461280; doi:10.1371/journal.pone.0214686)

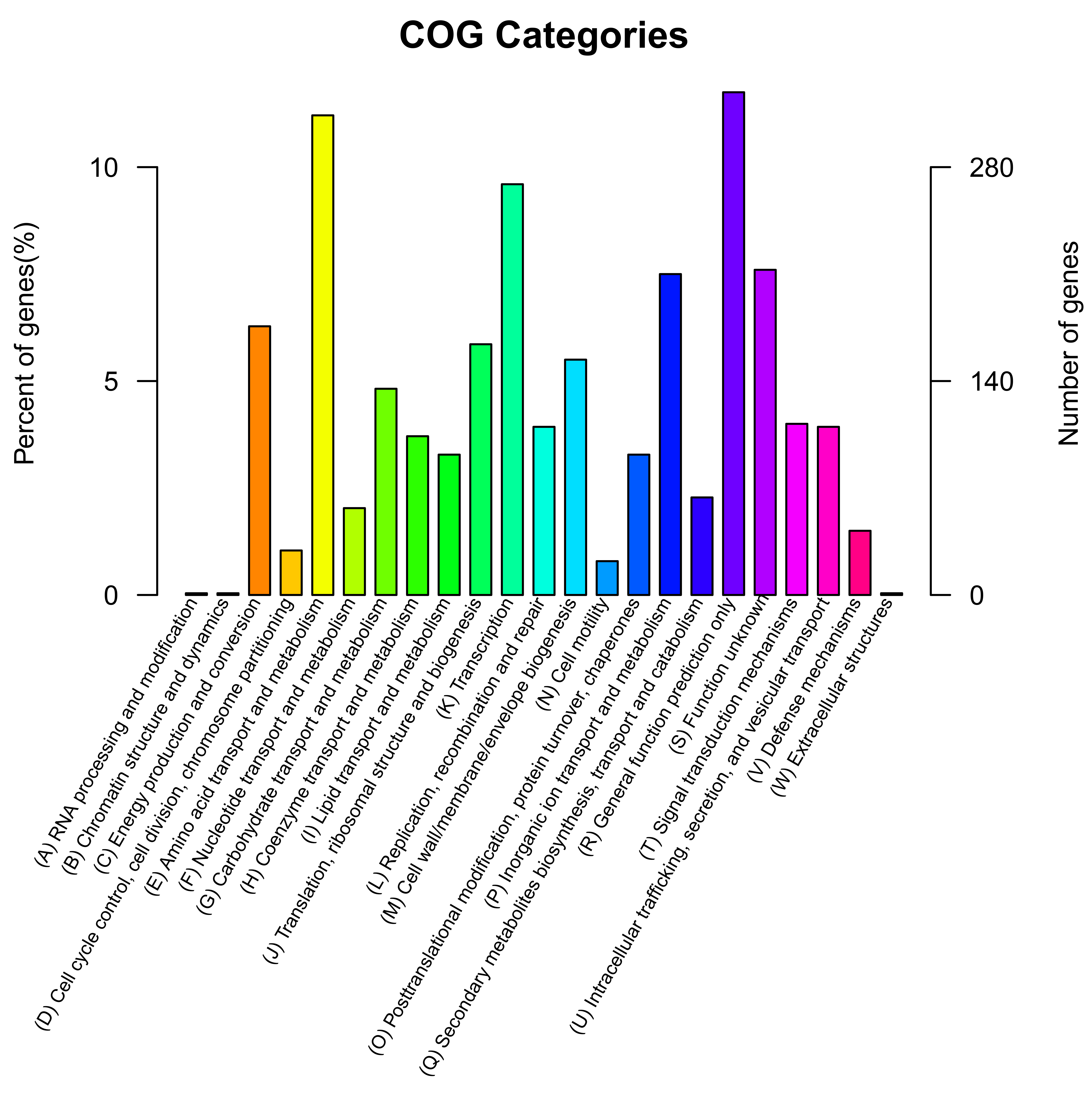

Supplement: S1 Fig — (TIF) [file pone.0214686.s001.tif]
